# Genotyping Performance between Saliva and Blood-Derived Genomic DNAs on the DMET Array: A Comparison

**DOI:** 10.1371/journal.pone.0033968

**Published:** 2012-03-20

**Authors:** Yueshan Hu, Erik A. Ehli, Kelly Nelson, Krista Bohlen, Christophina Lynch, Patty Huizenga, Julie Kittlelsrud, Timothy J. Soundy, Gareth E. Davies

**Affiliations:** 1 Avera Institute for Human Genetics, Sioux Falls, South Dakota, United States of America; 2 Department of Psychiatry, University of South Dakota, Sioux Falls, South Dakota, United States of America; 3 Avera Research Institute, Sioux Falls, South Dakota, United States of America; 4 Avera Cancer Institute, Sioux Falls, South Dakota, United States of America; 5 Department of Pharmaceutical Sciences, South Dakota State University, Brookings, South Dakota, United States of America; Karolinska Institutet, Sweden

## Abstract

The Affymetrix Drug Metabolism Enzymes and Transporters (DMET) microarray is the first assay to offer a large representation of SNPs conferring genetic diversity across known pharmacokinetic markers. As a convenient and painless alternative to blood, saliva samples have been reported to work well for genotyping on the high density SNP arrays, but no reports to date have examined this application for saliva-derived DNA on the DMET platform. Genomic DNA extractions from saliva samples produced an ample quantity of genomic DNA for DMET arrays, however when human amplifiable DNA was measured, it was determined that a large percentage of this DNA was from bacteria or fungi. A mean of 37.3% human amplifiable DNA was determined for saliva-derived DNAs, which results in a significant decrease in the genotyping call rate (88.8%) when compared with blood-derived DNAs (99.1%). More interestingly, the percentage of human amplifiable DNA correlated with a higher genotyping call rate, and almost all samples with more than 31.3% human DNA produced a genotyping call rate of at least 96%. SNP genotyping results for saliva derived DNA (n = 39) illustrated a 98.7% concordance when compared with blood DNA. In conclusion, when compared with blood DNA and tested on the DMET array, saliva-derived DNA provided adequate genotyping quality with a significant lower number of SNP calls. Saliva-derived DNA does perform very well if it contains greater than 31.3% human amplifiable DNA.

## Introduction

Genetic variation has been conclusively recognized as a critical contributor of individual therapeutic efficacy and/or side effects for any given drug. The Affymetrix Drug Metabolism Enzymes and Transporters (DMET) microarray is the first assay enabling the simultaneous genotyping of a large number of known markers (1,936 markers in 225 genes) in drug Absorption, Distribution, Metabolism & Excretion (ADME) [1]–[3]. The DMET array platform has been recently used by several research groups who have successfully identified new drug associated biomarkers [4], [5].

Blood samples have proven to be a gold standard source of genomic DNA for biomarker genotyping. However, the need to have a health professional draw the blood as well as the invasive character of this method significantly reduces participation rates [6], [7], and some study subjects such as psychiatric patients may be reluctant to provide blood samples [8]. The alternative is saliva-derived genomic DNA. The collection process is user-friendly, painless, and cost-effective. It is made more attractive by the availability of commercially available kits such as the Oragene·DNA kit [9]. There is concern, however, of point source microbial contamination inherent in the human saliva and how it may interfere with array genotyping call rates [10], [11] even though the human DNA could be specifically quantified by assaying for the human *RNase P* gene [12], [13].

Saliva has been reported to be a reliable source for DNA genotyping on the Affymetrix SNP 6.0 microarray platform (Scheet et al, unpublished data) and Illumina Hap370 microarray [14], but produces much lower genotyping call rates in Affymetrix Mapping 500 K Array [15] and even for some individual SNP assays [16]. The genotyping performance of saliva-derived DNA appears to be associated with the microarray type, presumably because of the different chemistries required to obtain the genotypes. To date, there are no such reports demonstrating the effect of DNA derived from human saliva on the genotyping performance for the DMET array, and also no comparisons have been made between blood and saliva derived DNA samples on this platform.

This study was designed to compare genotyping performance between blood and saliva-derived DNA on the DMET array. More importantly, the study also evaluated possible ways to improve the saliva-derived DNA genotyping call rate.

## Results

### The quantity and quality of genomic DNA extracted from both saliva and blood was adequate for the DMET array

We first compared the quantity and purity of isolated genomic DNA from both the blood and saliva samples. As shown in Table 1, the purity of genomic DNA extracted from the saliva samples is not significantly different than that from the blood samples. However, the DNA yield from saliva samples is significantly lower when compared to the blood samples.

**Table 1:** Comparison of DNA purity and yield between blood and saliva samples.

DNA Source	Purity (A260/A280)	Yield (µg)
Blood (n = 45)	1.85±0.004	253.63±26.6
Saliva (n = 42)	1.85±0.02	21.09±3.64
T-test P-value	0.709	1.32142×10^−11^

### Saliva-derived DNA contains significantly less human amplifiable DNA, and produces a significantly lower DMET genotyping call rate when compared with blood-derived DNA

The amplifiable human DNA from both blood and saliva-derived DNAs was determined using the Taqman RNase P assay. As shown in Table 2, the mean amplifiable human DNA percentage in saliva samples is significantly lower than that of the blood samples (37.3% vs. 87.57%). More interestingly, the genotyping call rate for the saliva-derived DNA is also significantly lower than blood-derived DNAs (88.82% vs. 99.1%), which is drastically lower than the desired genotyping call rate of 98% suggested by the manufacturer. The reduction in call rate correlates to an average of 1918/1936 markers called in the blood-derived samples compared to 1719/1936 SNPs called with the saliva-derived DNAs.

**Table 2:** Comparison of amplifiable human DNA percentage and genotyping call rate between blood and saliva derived DNA.

DNA Source	Amplifiable human DNA (%)	Genotyping call rate (%)
Blood (n = 45)	87.57±2.38	99.10±0.08
Saliva (n = 42)	37.3±4.2	88.82±1.83
T-test P-value	1.63828×10^−15^	1.64246×10^−6^

### The percentage of human amplifiable DNA in saliva samples is associated with the genotyping call rate

**Figure 1:**
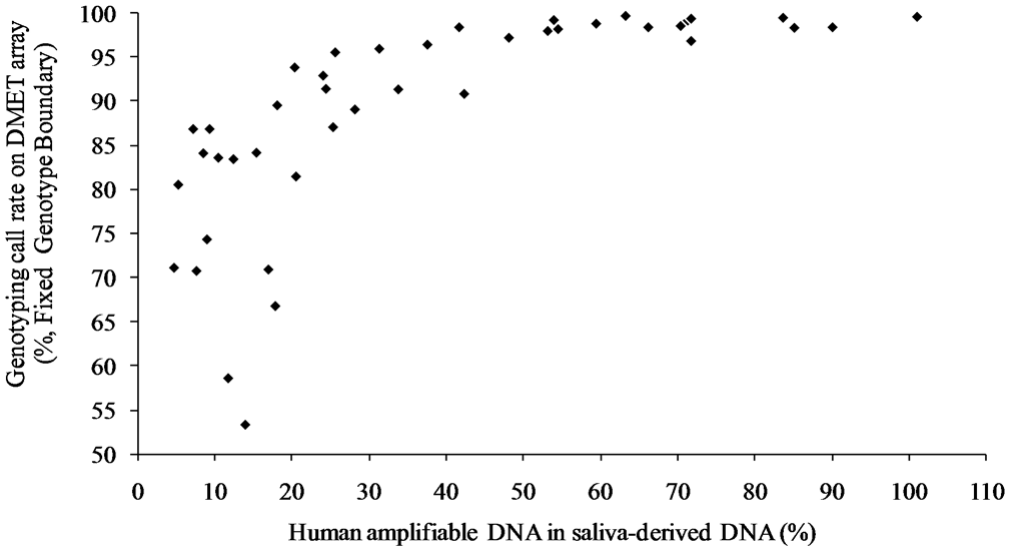
Association between the percentage of human DNA in saliva and the genotyping call rate of DMET arrays.

We evaluated the correlation of amplifiable human DNA percentage in saliva samples and their genotyping call rates on the DMET array. The results demonstrate that the genotyping call rates were enhanced as amplifiable human DNA percentage was increased (Figure 1). As a result, there is an association between the amplifiable human DNA and the genotyping call rate. Also, we found that most saliva samples (90%) containing  = 31.3% amplifiable human DNA had a genotyping call rate >96%.

### Increasing the concentration of saliva-derived DNA did not result in a significant enhancement of the genotyping call rate

Since a number of saliva-derived DNA samples demonstrated lower genotyping call rates, and this appeared to be associated with a lower amplifiable human DNA percentage, we hypothesized that increasing the saliva DNA concentration would increase the genotyping call rate by indirectly increasing the amount of human DNA. Therefore, we chose six samples and tested their performance on the DMET array after increasing the genomic DNA concentration (Table 3). The genotyping call rates did not increase or decrease when the human amplifiable DNA was changed. As a result, there is no significant difference of genotyping call rate between those samples with an increased concentration (average is 84.07±5.6%) when compared with the samples at their original concentration (84.83±3.61%) (P = 0.9041).

**Table 3:** DMET Genotyping call rate (%) following the increasein the concentration of saliva-derived DNA.

Sample #	Amplifiable Human DNA (%)	1× Conc.	1.3× Conc.	1.5× Conc.	1.6× Conc.	2× Conc.	2.7× Conc.	4× Conc.	8× Conc.	16× Conc.
1	5.2	80.58				77.94		90.01		
2	11.7	58.67				75.04				
3	15.4	84.21				90.89	92.91			
4	20.3	93.89		84.41		93.47				
5	24.4	91.46				85.19		86.95	80.06	64.73
6	25.6	95.6	91.77		89.38					

### Taqman SNP assay results confirm the genotyping quality of the DMET array

In order to evaluate the genotyping quality of markers on the DMET array, we randomly selected five SNPs included on the DMET marker panel, and subsequently genotyped the blood-derived DNA samples using Taqman SNP assays. When we compared the concordance of the genotyping results between the DMET array and the Taqman SNP assays, there was only one discordant genotype across all five SNPs and forty-five samples. The DMET array produced a 99.5% concordance when compared to a second platform (Allelic Discrimination Assay).

### The DMET genotyping concordance between saliva and blood-derived DNAs for all markers called on the DMET array is high

39 individuals provided both saliva and blood samples, we evaluated the genotyping concordance between the saliva and blood-derived genotype results. The data illustrated a 98.7% concordance across both tissues.

## Discussion

Peripheral blood samples have been the dominant DNA source for genotyping using individual SNP assays and next generation high density SNP microarrays [14], [16]. However, the requirement of a trained staff and the painful collection process significantly decreases the participation rate of patients [6], [7], especially for those with psychiatric diseases [8]. As a convenient, painless, and cost-effective alternative; saliva samples have been proven to be a good substitute to blood-derived DNAs for individual SNP assays [17] and for various platforms utilizing microarrays [14], [18]. However, the existence of exogenous point source microbial DNA in saliva-derived genomic DNA samples can significantly decrease the performance of the sample when genotyped on various array platforms [15], [16]. As a powerful research tool for pharmacogenomic studies, the DMET microarray provides the first high throughput platform to investigate typical pharmacokinetic markers at one time and the resulting allele translations greatly facilitate the use of these results in clinical applications [19], [20]. To our knowledge, this is the first time saliva-derived DNAs have been utilized for genotyping on the DMET array platform. We compared the genotyping performance of saliva-derived DNA against blood-derived DNA. In addition, we also have suggested a method to screen saliva-derived DNA to select samples that would perform optimally on this platform.

The DNA extraction results demonstrate that blood samples yield more DNA than saliva samples, which is mainly due to the utilization of a higher blood volume (8.5 ml) than saliva (0.5 ml) for extraction. Nevertheless, the saliva sample produced sufficient DNA (21 µg) in regard to the minimum amount required (1.05 µg) for DMET assays. The saliva DNA purity is very similar to that of blood DNA, but as spectrophotometry cannot differentiate the microbial DNA from human DNA, we utilized the RNase P TaqMan assay to identify amplifiable human DNA. The *RNase P* gene sequence is highly species-specific. The Homo sapiens RNase P assay only detects amplifiable human DNA instead of total genomic DNA. A limitation of this method for quantifying human DNA is the fact that DNA degradation and PCR reaction inhibitors may confound the results. Consequently, the amplifiable human DNA percentage determined for our blood samples is 87%. The saliva samples contain a significantly lower percentage of amplifiable human DNA (37.3%) when compared with blood samples.

Interestingly, saliva-derived DNA demonstrated a significantly lower genotyping call rate on the DMET array when compared with blood-derived DNA. The reasons for this difference in genotyping call rate may be explained by the unique character of genotyping the polymorphisms on this array. The assay involves the use of Molecular Inversion Probes (MIPs) [17], [21] which specifically recognize the SNPs, are PCR amplified, and hybridized to the probes on the DMET array. The existence of contaminating DNA in saliva samples may competitively interfere with binding of the MIPs to the target SNPs and adjacent sequences, preventing efficient target amplification. As a result, this could affect probe hybridization and ultimately have a negative impact on the genotyping call rate for these samples.

Our genotyping results illustrated an association between the percentage of amplifiable human DNA with the saliva DNA genotyping call rate. The majority (18/20) of saliva samples containing  = 31.3% of amplifiable human DNA had a genotyping call rate greater than 96%. Two samples containing 33.7% and 42.3% of human amplifiable DNA had a genotyping call rate of 91.4% and 90.89% respectively. This is consistent with another study indicating poor genotyping performance utilizing saliva-derived DNA with less than 30% of human amplifiable DNA on the Affymetrix 500 K GeneChip platform [15]. These results provide a screening method (RNase P assay) and threshold value (31.3%) for future research groups interested in genotyping saliva samples on the DMET array.

One goal of any molecular genetics researcher using the DMET array is to obtain the most SNP genotype calls for their samples. At this time, there is not a unified accepted standard for the genotyping call rate on the DMET platform. A manufacturer suggested threshold of 98% represents a quantity of SNP genotypes called from the array (1897/1936), but does not infer the quality of genotypes called. The results from this comparison study are not meant to propose a new threshold (96%) for the genotyping call rate when performing clinical or research studies. The results from this study are to simply guide researchers considering the use of saliva-derived genomic DNA in DMET studies, using a threshold of human amplifiable DNA as a percentage in the sample which provides an indication that these will then perform adequately (1857/1936 SNPs called) on the DMET array. As a result, the researchers can screen their samples before proceeding onto the more expensive experiment.

In attempts to improve the genotyping call rate for saliva samples containing <31.3% human amplifiable DNA, we increased the concentration of input genomic DNA for the assay. This intervention indirectly increases the absolute amount of human amplifiable DNA required for the assay however, this did not significantly change the genotyping call rate (84.1% vs. 84.8%). We hypothesize that the concomitant increase in contaminating DNA may also inherently interfere with genotyping performance.

The results from our Taqman SNP assays confirmed the excellent genotyping quality of the DMET array platform (99.5% reproducibility). The concordance check of genotyping calls between saliva and blood derived DNAs, also illustrated a very high percentage (98.7%) of SNPs with the same genotype, demonstrating the high quality of calls for saliva samples even in the samples in which the human DNA percentage is low. This suggests that the contaminating DNA in saliva does not affect the quality of the genotyping calls, but only has an effect on the number of markers that are ultimately genotyped.

In conclusion, genomic DNA extracted from saliva and blood produced high quality genotyping results on the DMET platform. However, the number of markers called using saliva-derived DNA was significantly less than that of blood-derived DNA. We have presented a threshold (31.3%), for the percentage of human amplifiable DNA in the saliva genomic DNA prep, which will produce a genotyping call rate of at least 96%. This has important implications to other research groups wishing to utilize the DMET platform, especially if there are significant barriers to collecting blood for a genomic DNA source or when saliva has already been collected many years ago and biobanked.

## Materials and Methods

### DNA extraction

Saliva samples (n = 42) were collected from patients with mental disorders at a South Dakota Developmental Center with ORAgene·DNA vials (DNA GenoTek, Kanata, Ontario Canada). Genomic DNA from 500 µl of the oragene/saliva mix was extracted with Oragene·DNA purifier following the manufacturer's protocol. The concentration of the DNA sample was determined using the NanoDrop ND-1000 spectrophotometer (NanoDrop Technologies, Wilmington, DE USA) and the DNA yield was calculated by multiplying by the volume. The A260/A280 ratio was used to evaluate DNA purity.

Peripheral blood samples (n = 45) were collected with PAXgene™ blood DNA tubes (Qiagen, Valencia, CA USA) prefilled with DNA stabilizing reagents. 39 individuals provided both a blood and saliva sample. The tube containing 8.5 ml whole blood was stored at 4°C for less than 4 weeks and DNA was extracted with the PAXgene ™ blood DNA kit (Qiagen, Valencia, CA USA) following the manufacturer's protocol. DNA purity and quantity were checked using spectrophotometry using the methods described above.

The study protocol was approved by the Institutional Review Board (IRB) of Avera McKennan Hospital. The guardian, whether self or Legally Authorized Representative (LAR), signed the informed consent and when appropriate the subject signed Assent. The Assent was obtained from those subjects who had the mental capacity to agree to be in the study but did not hold their own guardianship. The guardian, if not self, was a family member who was assigned as the LAR or state assigned guardian.

### RNase P assay

The Taqman RNase P Assay (Life Technologies, Carlsbad, CA USA) was used to measure the amount of human amplifiable DNA in both the saliva and blood-derived DNA samples. A standard curve was established using the human genomic control DNA supplied in the kit, upon which the amount of human DNA in samples are calculated. Real-time PCR was performed following the manufacturer's protocol. Briefly, samples were assayed using 384-well plates. To each well, 5 µl TaqMan Universal PCR Master Mix, 0.5 µl RNase P primer-Probe Mix, 0.5 µl water, and 4 µl template DNA (10 ng/µl) was added. The PCR reaction was performed on the Life Technologies 7900HT Fast Real-Time PCR System using standard reaction conditions. The human DNA percentage was determined by dividing the amount of human DNA (as determined by the Taqman Assay) by the total amount of DNA added to the reaction.

### DMET assay

42 saliva-derived DNA samples and 45 blood-derived DNA samples were run on the DMET microarray using the DMET Plus Premier pack kits according to the protocol described in the DMET Plus Premier pack User Guide. Briefly, some markers from regions containing pseudogenes and close homologs are first pre-amplified using a multiplex polymerase chain reaction (mPCR) (Qiagen, Valencia, CA USA). Genomic sequences that contain the polymorphic markers of interest are preferentially amplified through the use of highly selective molecular inversion probes (MIPs). A first quality control (QC) gel is run to determine the quality of amplified MIPs, which should be a single band represented on a gel in the range of 100–150 base pairs. Smaller DNA fragments were generated by adding fragmentation reagents to improve sample hybridization with the DMET plus array, and DNA fragment size is checked on the second QC gel, in which the fragments length should be less than 120 base pairs with a smear centered at approximately 50 base pairs. Fragments were labeled using the supplied labeling reagents and then hybridized to the DMET microarray at 49°C for 16–18 hours in the Affymetrix hybridization oven rotating at 35 rpm. Hybridized DMET arrays were washed and stained in the Affymetrix fluidic stations and scanned with the Affymetrix GeneChip® Scanner 3000 7G. Genotyping data was generated with Affymetrix GeneChip® Command console software and analyzed with the DMET Console software.

### TaqMan SNP assays

With the aim of confirming the reproducibility of genotyping results produced by DMET assay, we chose to perform 195 tests by selecting five SNPs (Single Nucleotide Polymorphism) from the DMET marker panel and test 39 samples for genotype concordance using TaqMan SNP assays in the Life Technologies 7900HT Fast Real-Time PCR System. 195 tests would enable the detection of a 0.5% change in concordance between the two platforms, as Affymetrix reports a 99.5% concordance to reference. The five SNPs (rs56107638, rs2470890, rs3892097, rs28371725, and rs762551) were selected due to their performance on the DMET array when performing saliva genotyping. The genotyping call rates (35.9%, 66.7%, 69.2%, 82.1%, 89.7% respectively) for each of the five SNPs were less than or equal to the mean call for all the SNPs (1936) when genotyped on the DMET array (88.8%) using 39 saliva samples. The dbSNP ID, DMET probe ID, and assay probe context sequence for each of the SNPs tested in this experiment are listed in Table 4. The assays were ordered from Life Technologies and real-time PCR was performed using 2.5 µl 2X Taqman genotyping master mix, 0.125 µl 40X primer-probe mix, 1.375 µl water and 1 µl of DNA sample (10 ng/ µl). PCR cycling conditions consisted of a 10 min denaturation at 95°C and 40 cycles of 95°C for 15 sec and 60°C for 1 min. The post assay analysis was performed using the Life Technologies SDS (version 1.3) software.

**Table 4:** SNP primer-probe sets ordered from Life Technologies.

DMET probe ID	dbSNP ID	Context Sequence [VIC/FAM]
AM_10807	rs2470890	AGGCGCGGCTGCGCTTCTCCATCAA[T/C]TGAAGAAGACACCACCATTCTGAGG
AM_10785	rs762551	TGCTCAAAGGGTGAGCTCTGTGGGC[C/A]CAGGACGCATGGTAGATGGAGCTTA
AM_10802	rs56107638	ACCAGTGGCAGGTCAACCATGACCC[A/G]TGAGTACATACCCCTCACGAAAAAA
AM_12274	rs3892097	AGACCGTTGGGGCGAAAGGGGCGTC[C/T]TGGGGGTGGGAGATGCGGGTAAGGG
AM_12257	rs28371725	TTCATGGGCCCCCGCCTGTACCCTT[C/T]CTCCCTCGGCCCCTGCACTGTTTCC

### DMET Console analyses

Genotyping call rate and concordance comparisons were analyzed using the DMET Console (version 1.2) software. Fixed Genotype Boundaries was used as the algorithm for all genotyping configurations. The recommended QC call rate (same value as genotyping call rate in DMET array) threshold is greater than 98%. Genotyping results from 39 patients with both saliva and blood-derived DNAs were evaluated for SNP concordance across both tissues.

### Statistical analysis

Two-tailed Student's t-tests were used to evaluate the differences between saliva and blood-derived DNA samples. Significant difference was defined as p = 0.01.
